# Identification of potential miRNA biomarkers for neurobrucellosis diagnosis

**DOI:** 10.3389/fphys.2025.1463597

**Published:** 2025-06-23

**Authors:** Meiling Liu, Liping Feng, Shujun Shi, Yuzhou Guo, Menghan Lv, Zhengyu Li, Xintian Wang, Hao Yang, Zhelin Zhang

**Affiliations:** ^1^ Department of Neurology, The Affiliated Hospital of Inner Mongolia Medical University, Hohhot, China; ^2^ Xing’an League Disease Prevention and Control Center, Ulanhot, China; ^3^ Department of Radiation Oncology, Inner Mongolia Cancer Hospital & Affiliated People’s Hospital of Inner Mongolia Medical University, Hohhot, China

**Keywords:** neurobrucellosis, miRNAs, cerebrospinal fluids, miR-499a-5p, miR-576-5p

## Abstract

**Background:**

Neurobrucellosis (NB) is a rare complication of brucellosis that presentis with a variety of clinical symptoms, complicating its diagnosis. This study aimed to analyze the microRNA (miRNA) profile of cerebrospinal fluid (CSF) in NB patients to identify potential biomarkers for NB diagnosis.

**Methods:**

Small RNA sequencing was utilized to identify differential expressed miRNAs (DEmiRNAs) in CSF samples from both control and NB patients (in-house cohort). The GSE107554 dataset (validation cohort) from the GEO database was used to validate the expression levels of these DEmiRNAs. Subsequently, LASSO regression analysis was conducted to construct a diagnostic risk score model based on common miRNAs identified in both the in-house and validation cohorts.

**Results:**

The study identified 51 DEmiRNAs between the control and NB groups, based on the data from the in-house cohort. Among these, four common miRNAs (miR-342-3p, miR-576-5p, miR-15b-5p and miR-499a-5p) were significantly elevated in the infection group compared to controls in both the in-house and validation cohorts. Subsequently, a novel NB diagnostic signature comprising two miRNAs (miR-576-5p and miR-499a-5p) was developed using the LASSO regression analysis. Receiver operating characteristic (ROC) analysis demonstrated the good diagnostic potential of this miRNA signature in both cohorts. RT-qPCR analysis indicated elevated levels of miR-576-5p and miR-499a-5p in CSF samples of NB patients compared to controls. Additionally, RT-qPCR results revealed decreased levels of VAV3 (vav guanine nucleotide exchange factor 3, a target of miR-499a-5p) and IGF1 (insulin like growth factor 1, a target of miR-576-5p) in the CSF samples of NB patients.

**Conclusion:**

Collectively, miR-576-5p and miR-499a-5p may serve as potential biomarkers for the diagnosis of NB.

## 1 Introduction

Brucellae are Gram-negative bacteria that infect mammals and can result in brucellosis (Glowacka et al., 2018). Brucellosis is the most widespread zoonosis globally (Hull and Schumaker, 2018), posing a serious threat to human health while also affecting the advancement of animal husbandry. Clinical symptoms of human brucellosis include excessive sweating, fever, joint and muscle pain, and inflammatory diseases such as sacroiliitis, arthritis, and osteomyelitis (Yang et al., 2019). If left untreated, brucellosis can result in severe health complications that are difficult to treat (Pritam and Kumar, 2024). Annually, approximately 500,000 cases of human brucellosis are reported globally (Samadi et al., 2024; Pappas et al., 2006). Timely diagnosis and treatment of brucellosis can greatly improve patient outcomes.

Neurobrucellosis (NB) is a severe focal infection caused by *Brucella* bacteria (Alhatou et al., 2024). A significant feature of NB is neuritis, which is characterized by inflammation of the nerves, and can present as meningitis, optic neuritis, peripheral neuritis, and myelitis (Qasim et al., 2021; Havali and Cagan, 2020; Mantur et al., 2004). Additionally, a case study by Vinod et al. reported a rare instance of NB in a patient exhibiting symptoms such as vision loss, retrobulbar neuritis, headache, and left hemiparesis (Vinod et al., 2007). However, the pathogenesis of NB remains poorly understood, and there are no definitive diagnostic criteria available. Clinical symptoms, imaging findings and cerebrospinal fluid (CSF) tests are often atypical for NB, leading to confusion with other neurological conditions and potentially resulting in missed or incorrect diagnoses (Cao et al., 2022; Kizilkilic and Calli, 2011). Thus, it is crucial to identify reliable biomarkers for the accurate diagnosis of NB.

MicroRNAs (miRNAs), which are small endogenous RNAs, can modulate gene expression post-transcriptionally (Ho et al., 2022; Qian et al., 2023). Research has demonstrated that changes in miRNA expression can lead to modifications in gene profile, thereby affecting various biological processes and contributing to the development of multiple human disorders (Ho et al., 2022). Given their high stability in human fluids, circulating miRNAs are being explored as potential biomarkers for disease diagnosis (Ho et al., 2022).

CSF analysis serves as a crucial diagnostic tool for various conditions affecting the central nervous system (CNS), particularly CNS infectious diseases (Gomes, 2022). The CSF directly interacts with both the brain and spinal cord, forming a protective environment that is essential for the proper functioning of these CNS structures (Panyard et al., 2021). It is important to note that the CSF is separated from the bloodstream by the blood-brain barrier, this unique positioning enables the CSF to provide a more immediate and accurate representation of the physiological changes occurring within the CNS compared to other types of biological samples (Panyard et al., 2021). Yang et al. discovered that certain metabolites in CSF samples could potentially serve as biomarkers for distinguishing patients with NB from controls (Yang et al., 2022). This study highlights the significance of CSF as a valuable biological fluid for exploring new biomarkers for NB. The objectives of our study were to identify potential biomarkers for the diagnosis of NB, which could play a significant role in diagnosing NB.

## 2 Subjects and methods

### 2.1 Sample collection

This study included a total of 19 patients with external hydrocephalus (Control group) and 19 patients with NB from Inner Mongolia Cancer Hospital & Affiliated People’s Hospital of Inner Mongolia Medical University were included in this study. The clinical manifestations of these patients were presented in Supplementary Table S1. CSF samples (5 mL per sample) were collected by lumbar puncture from all patients, respectively. All samples were immediately snap-frozen in liquid nitrogen. Prior to participation, all individuals provided written informed consent and the study received approval from the biomedical research ethics committee of Inner Mongolia Medical University (No. YKD202202045). The study was conducted in accordance with the Declaration of Helsinki.

The inclusion criteria for patients with NB were as follows: (1) individuals exhibiting signs and symptoms consistent with NB; (2) *Brucella* species were isolated from CSF samples and/or anti-Brucella antibody was detected in CSF samples; (3) individuals presenting with elevated protein levels, and decreased glucose levels in the CSF and lymphocytosis; and (4) brain magnetic resonance imaging (MRI) or computed tomography (CT) scans were performed support the diagnosis of NB. The exclusion criteria for patients with NB included the following: (1) patients with other bacterial or viral infections affecting the CNS; (2) patients who received antibiotic therapies before the collection of CSF; (3) patients with other metabolism-related encephalopathies; (4) patients who refused lumbar puncture; (5) patients with coagulation disorders; (6) patients with hepatic and renal insufficiencies or severe underlying cardiopulmonary diseases.

The inclusion criteria for patients with hydrocephalus included: (1) patients exhibiting significant enlargement of the ventricular system or periventricular interstitial edema, consistent with the imaging diagnosis of hydrocephalus; (2) patients presenting with gait disorders, cognitive dysfunction, voiding dysfunction, upper limb hypermobility, or other clinical signs; (3) patients demonstrating a positive CSF drainage test. The exclusion criteria for patients with hydrocephalus included: (1) patients with intracranial infections; (2) patients with other metabolism-related encephalopathies; (3) patients who refused lumbar puncture; (4) patients with coagulation disorders; (5) patients with hepatic and renal insufficiencies or severe underlying cardiopulmonary diseases.

The CSF samples from four patients in the control group and four patients in the NB group were used for small RNA-sequencing. Subsequently, the CSF samples from 15 patients in the control group and 15 patients in the NB group were subjected to real-time quantitative polymerase chain reaction (RT-qPCR) assay to validate the expression levels of the hub miRNAs between the control and NB groups.

### 2.2 Small RNA-sequencing

Total RNA was extracted from the CSF samples using the TRIzol reagent. Subsequently, the NEBNext Small RNA Library Prep Set for Illumina kit (NEB#E7330S, NEB) was used for constructing the small RNA library. Small RNA sequencing was then performed on the Illumina NovaSeq 6000 sequencing platform. Following the acquisition of raw data from small RNA-sequencing, clean reads were obtained by removing low-quality reads. Using Bowtie software, the clean reads were compared with the data in the Rfamv.10.1 (http://www.sanger.ac.uk/software/Rfam) to annotate and filter ribosomal RNA (rRNA), small cytoplasmic RNA (scRNA), small nuclear RNA (snRNA), transfer RNA (tRNA) and other RNA types. The reads were then compared with the cDNA sequence, Repbase database and miRBase v22 database (http://www.mirbase.org/) using Bowtie software to identify and annotate known mature miRNAs. Meanwhile, the expression patterns of miRNAs were then analyzed across different samples. Next, novel miRNAs were identified by processing unannotated reads using miRDeep2.

### 2.3 Differential expression analysis

Differentially expressed miRNAs (DEmiRNAs) between the control group (n = 4) and NB group (n = 4) were identified using the “limma” function package in R language (version 3.56.2) with thresholds of |Log2fold change|>1 and false discovery rate (FDR) < 0.05 (Ritchie et al., 2015; Lu et al., 2021).

### 2.4 Functional enrichment analysis

Two online databases, Targetscan (https://www.targetscan.org/vert_80/) and miTarBase (http://mirtarbase.mbc.nctu.edu.tw/index.html), were used to predict the target genes of DEmiRNAs. Gene ontology (GO) and Kyoto Encyclopedia of Genes and Genomes (KEGG) functional analyses were performed on these target genes using the “clusterProfiler” function package in R language, with a significance threshold set at p-value <0.05.

### 2.5 Data acquisition

The GSE107554 dataset, which includes 23 peripheral blood CD4 T cell samples from 15 brucellosis patients and 8 healthy controls, was downloaded from Gene Expression Omnibus (GEO, https://www.ncbi.nlm.nih.gov/geo/) database. This dataset was set as a validation cohort.

### 2.6 LASSO regression analysis

LASSO Cox regression analysis with was conducted using the “glmnet” package in R language to further identify miRNAs associated with NB (Friedman et al., 2010). The risk score for each sample was calculated using the formula: risk score = $\sum_{i=1}^{n}x_{i}*Coef_{i}$ . Xi represents the expression value of each miRNA, Coefi denotes the risk coefficient of each miRNA as determined by the LASSO-Cox model.

### 2.7 Construction of miRNA-mRNA regulatory network

Targetscan and miTarBase databases were used to predict the downstream target genes of miR-499a-5p and miR-576-5p. The cytoscape software (version 3.7.2) was used to visualize the miRNA-mRNA regulatory network (Shannon et al., 2003).

### 2.8 RT-qPCR

The Redzol reagent (No. FTR-50, SBS Genetech Co., Ltd) was used for the extraction of total RNA. Next, the extracted RNA was reverse-transcribed into cDNA using the SureScript™ First-Strand cDNA Synthesis Kit (No. QP056, GeneCopeia). After that, qPCR was conducted using the 2×SYBR Green qPCR Master Mix (None ROX) kit (No. MPC2203026, Servicebio). The relative levels of vav guanine nucleotide exchange factor 3 (VAV3) and insulin like growth factor 1 (IGF1) were normalized to GAPDH, and the relative levels of miR-576-5p and miR-499a-5p were normalized to U6, and relative gene expression was calculated uisng the 2^−ΔΔCT^ method (Qian et al., 2020). The primers used in this study were provided as follows: GAPDH: forward, TGACTTCAACAGCGACACCCA and reverse, CACCCTGTTGCTGTAGCCAAA; U6: forward, CTCGCTTCGGCAGCACA and reverse, AACGCTTCACGAATTTGCGT; miR-499a-5p: forward, GACACGGCTCCGTCAC and reverse, ATCCCTGATCAACTGTCCGCC; miR-576-5p: forward, GCGCGATTCTAATTTCTCCAC and reverse, AGTGCAGGGTCCGAGGTATT; VAV3: forward, TGCGAAGAACTCCTAAAC and reverse, TTGGCAAGAATAATCTACTG; IGF1: forward, AGCAGTCTTCCAACCCAA and reverse, ATACATCTCCAGCCTCCTTA.

### 2.9 Luciferase reporter assay

The 3′-UTR fragments of VAV3 consisting of wild-type (WT) or mutant (Mut) binding site for miR-499a-5p was cloned into pmiRGLO (Tianjin Hongke Biotechnology Co., LTD.) to generate pmiRGLO-VAV3-WT/Mut luciferase reporter vectors. Next, 293T cells were co-transfected with pmiRGLO-VAV3-WT- or -Mut-3′UTR and miR-499a-5p mimics, mimics NC, ASO-miR-499a-5p or ASO-NC for 48 h using Lipofectamine2000 Reagent.

The 3′-UTR fragments of IGF1 consisting of wild-type (WT) or mutant (Mut) binding site for miR-576-5p was cloned into pmiRGLO (Tianjin Hongke Biotechnology Co., LTD.) to generate pmiRGLO-IGF1-WT/Mut luciferase reporter vectors. Next, 293T cells were co-transfected with pmiRGLO-IGF1-WT- or -Mut-3′UTR, and miR-576-5p mimics, mimics NC, ASO-miR-576-5p or ASO-NC for 48 h using Lipofectamine2000 Reagent. Subsequently, the luciferase activity was detected using the dual luciferase reporter gene detection kit (RG009, Beyotime).

### 2.10 Statistical analysis

Pearson correlation coefficients were calculated using the R language. Wilcoxon rank-sum test was employed to compare differences between groups. Receiver operating characteristic (ROC) curves were generated using the “pROC” package (version 1.18.4) in R language (Obuchowski and Bullen, 2018). All statistical analyses and graphics based on the data from in-house cohort and the validation cohort (GSE107554 dataset) were conducted using the R language (version 4.3.2). For RT-qPCR analysis, all graphs were created using GraphPad Prism software (version 9.5.0), and the data were also statistically analyzed using GraphPad Prism, with an unpaired t-test applied to assess statistical significance. For the results of luciferase reporter assay, two-way ANOVA was utilized to assess statistical significance. Difference was considered statistically significant when the p value was <0.05.

## 3 Results

### 3.1 Identification of DEmiRNAs between control and NB groups

**FIGURE 1:**
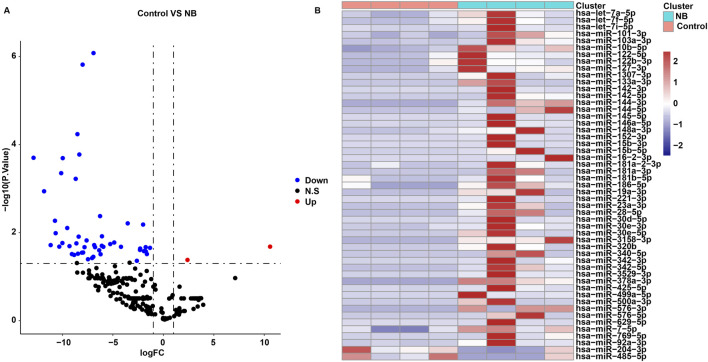
Identification of DEmiRNAs between control and NB groups. **(A)** Volcano plots and **(B)** heatmap showed DEmiRNAs between control and NB groups in the in-house cohort.

The “Limma” package was used to identify DEmiRNAs between the control and NB groups. A total of 51 DEmiRNAs were identified between two groups (Supplementary Table S2; Figures 1A, B). Compared to the NB group, 49 miRNAs were significantly downregulated and 2 miRNAs were notably upregulated in the control group (Supplementary Table S2; Figures 1A, B).

Next, we predicted the target genes of 51 DEmiRNAs using Targetscan and miTarBase databases. Among the 49 miRNAs that were downregulated in the control group, 44 miRNAs had common target genes between the two databases (Supplementary Table S3; Supplementary Figure S1). However, the target genes of the 2 miRNAs that were upregulated in the control group were predicted by the TargetScan database only (Supplementary Table S3; Supplementary Figure S2).

**FIGURE 2:**
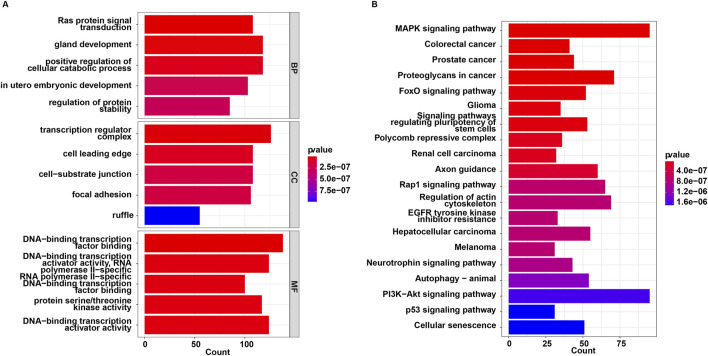
Functional enrichment analysis for target genes of 46 miRNAs. **(A)** GO and **(B)** KEGG enrichment analysis.

The 44 downregulated miRNAs and 2 upregulated miRNAs had 3,018 non-duplicated target genes. GO and KEGG analysis were then performed on these target genes. These target genes were enriched in 1003 GO terms including “Ras protein signal transduction”, “transcription regulator complex” and “DNA-binding transcription factor binding”, and were involved in 121 KEGG pathways such as “MAPK signaling pathway”, “FoxO signaling pathway”, “autophagy” and “PI3K-Akt signaling pathway” (Supplementary Table S4; Figures 2A, B).

### 3.2 Screening candidate miRNAs related to NB

**FIGURE 3:**
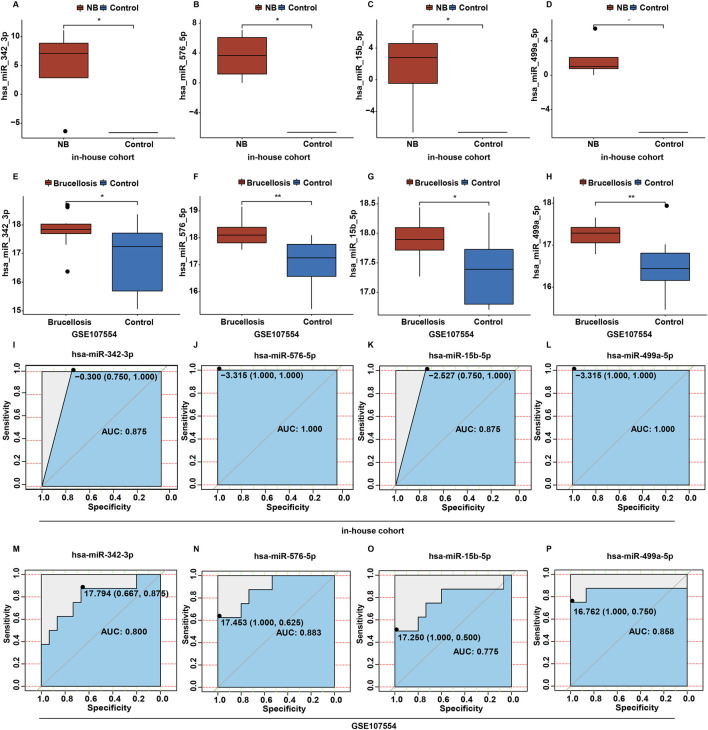
Screening candidate miRNAs related to NB. **(A–D)** The box plots of hsa-miR-342-3p, hsa-miR-576-5p, hsa-miR-15b-5p and hsa-miR-499a-5p levels between control and NB group in the in-house cohort. **(E–H)** The box plots of hsa-miR-342-3p, hsa-miR-576-5p, hsa-miR-15b-5p and hsa-miR-499a-5p levels between control and brucellosis group in the validation cohort. **(I–L)** Based on the data in the in-house cohort, ROC curves constructed to evaluate the diagnostic accuracy of hsa-miR-342-3p, hsa-miR-576-5p, hsa-miR-15b-5p and hsa-miR-499a-5p for NB. **(M–P)** Based on the data in the validation cohort, ROC curves constructed to evaluate the diagnostic accuracy of hsa-miR-342-3p, hsa-miR-576-5p, hsa-miR-15b-5p and hsa-miR-499a-5p for brucellosis.

The GSE107554 dataset was utilized to validate the expression levels of 51 DEmiRNAs and identify potential biomarkers in NB. As shown in Figures 3A–H, the expression profiles of four miRNAs (hsa-miR-342-3p, hsa-miR-576-5p, hsa-miR-15b-5p and hsa-miR-499a-5p) were consistent in both the in-house and validation cohorts. Compared to the control group, the levels of miR-342-3p, miR-576-5p, miR-15b-5p and miR-499a-5p were significantly upregulated in CSF samples from NB patients in the in-house cohort (Figures 3A–D). Receiver operating characteristic (ROC) analysis was used to assess the diagnostic efficiency of these four miRNAs in NB. In the in-house cohort, when the AUC of miR-342-3p was 0.875 and the cutoff value was −0.300, the specificity was 0.750 and the sensitivity was 1.000 (Figure 3I); when the AUC of miR-576-5p was 1.000 and the cutoff value was −3.315, the specificity was 1.000 and the sensitivity was 1.000 (Figure 3J); when the AUC of miR-15b-5p was 0.875 and the cutoff value was −2.527, the specificity was 0.750 and the sensitivity was 1.000 (Figure 3K); when the AUC of miR-499a-5p was 1.000 and the cutoff value was −3.315, the specificity was 1.000 and the sensitivity was 1.000 (Figure 3L).

In the validation cohort, when the AUC of miR-342-3p was 0.800 and the cutoff value was 17.794, the specificity was 0.667 and the sensitivity was 0.875 (Figure 3M); when the AUC of miR-576-5p was 0.883 and the cutoff value was 17.453, the specificity was 1.000 and the sensitivity was 0.625 (Figure 3N); when the AUC of miR-15b-5p was 0.775 and the cutoff value was 17.250, the specificity was 1.000 and the sensitivity was 0.500 (Figure 3O); when the AUC of miR-499a-5p was 0.858 and the cutoff value was 16.762, the specificity was 1.000 and the sensitivity was 0.750 (Figure 3P).

In two cohorts, the AUC of ROC curves for miR-342-3p, miR-576-5p and miR-499a-5p were greater than 0.80, suggesting that these three miRNAs had good diagnostic performances (Mandrekar, 2010). Meanwhile, the AUC of ROC curve for miR-15b-5p were greater than 0.70 in two cohorts, suggesting that miR-15b-5p had acceptable diagnostic performance (Mandrekar, 2010). These findings suggested that these four miRNAs had certain to good diagnostic performances.

### 3.3 Construction a diagnostic risk score model

**FIGURE 4:**
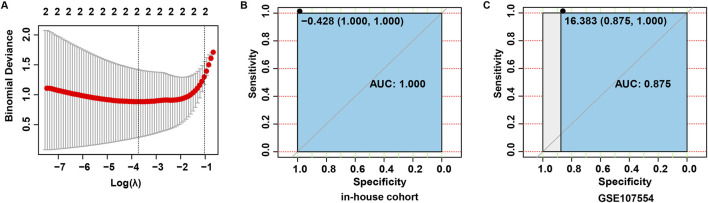
Construction a diagnostic risk score model. **(A)** Screening for tuning parameter (lambda) in the LASSO regression model. **(B)** Based on the data in the in-house cohort, ROC curves constructed to evaluate the diagnostic accuracy of the risk score for NB. **(C)** Based on the data in the validation cohort, ROC curves constructed to evaluate the diagnostic accuracy of the risk score for brucellosis.

To construct a diagnostic risk gene signature associated with NB, these four candidate miRNAs were subjected to the LASSO regression analysis. Based on the lambda values obtained from the LASSO regression analysis, the optimal number of miRNAs was determined to be 2 (Figure 4A). Subsequently, 2 optimal miRNAs, miR-576-5p and miR-499a-5p, were identified as central miRNAs related to NB. Next, the LASSO regression coefficients were weighted with miRNA levels to calculate the risk score using the formula: risk score = (0.2177862 × miR-576-5p) + (0.620704 × miR-499a-5p). The predictive ability of the risk score was then assessed by determining the area under the curve (AUC) value of the ROC curve. As shown in Figures 4B, C, the AUC value of the ROC curve was 1.000 in the in-house cohort, and was 0.875 in the validation cohort, indicating that the risk score may have a good diagnostic performance.

### 3.4 Functional enrichment analysis

**FIGURE 5:**
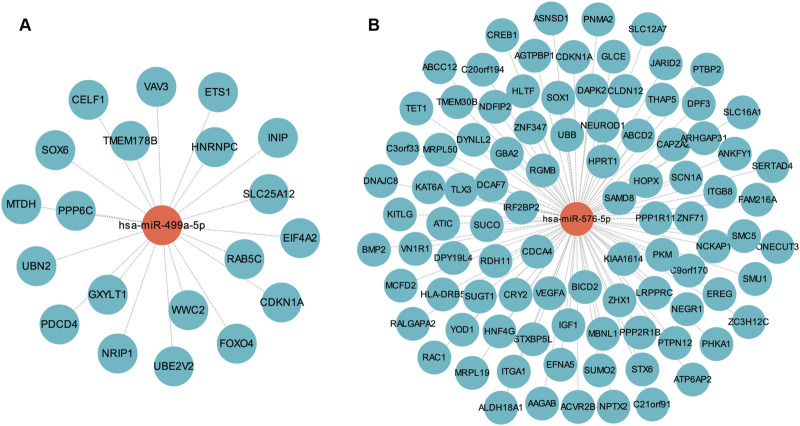
MiRNA-mRNA regulatory network. **(A)** The network of miR-499a-5p and its target genes. **(B)** The network of miR-576-5p and its target genes.

**FIGURE 6:**
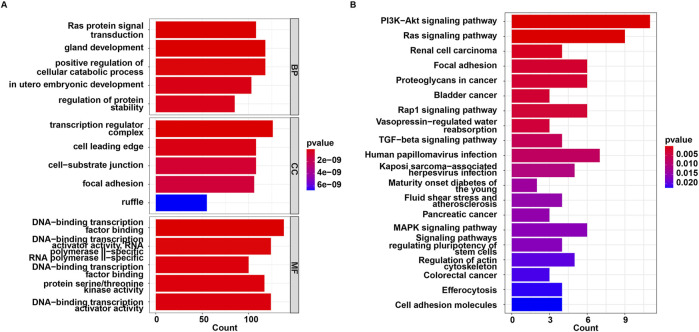
Functional enrichment analysis for target genes of miR-576-5p and miR-499a-5p. **(A)** GO and **(B)** KEGG enrichment analysis.

Based on data from the Targetscan and miTarBase databases, miR-576-5p was found to target 94 genes, and miR-499a-5p targeted 20 genes (Figures 5A, B; Supplementary Table S5). MiR-576-5p and miR-499a-5p were found to have 113 un-repeated target genes. GO and KEGG analysis were then performed on these 113 genes. These genes were enriched in 357 GO terms including “Ras protein signal transduction”, “transcription regulator complex” and “DNA-binding transcription factor binding”, and were involved in 30 KEGG pathways such as “PI3K-Akt signaling pathway”, “Ras signaling pathway” and “TGF-beta signaling pathway” (Supplementary Table S6; Figures 6A, B).

### 3.5 Validation of miR-499a-5p, miR-576-5p, VAV3 and IGF1 levels in NB

**FIGURE 7:**
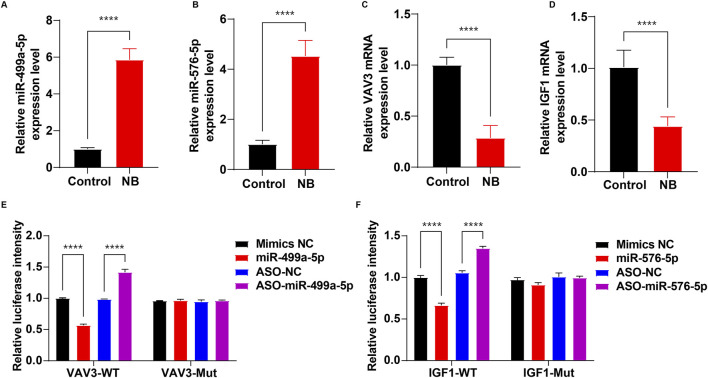
Validation of miR-499a-5p, miR-576-5p, VAV3 and IGF1 levels in NB. **(A–D)** RT-qPCR was used to determine the levels of miR-499a-5p, miR-576-5p, VAV3 and IGF1 in CSF samples between control and NB groups (n = 15 per group). **(E)** The luciferase activity assay to detect the targeting of VAV3 and miR-499a-5p. **(F)** The luciferase activity assay to detect the targeting of IGF1 and miR-576-5p. ****P < 0.0001.

Next, RT-qPCR was performed to validate the expression levels of miR-499a-5p and miR-576-5p, as well as their target genes VAV3 and IGF1 in CSF samples from control and NB patients, respectively. As shown in Figures 7A–D, compared to the control group, the levels of miR-499a-5p and miR-576-5p were notably upregulated, and the levels of VAV3 and IGF1 were remarkably declined in CSF sample of NB patients.

To determine whether VAV3 is a target of miR-499a-5p and IGF1 is a target of miR-576-5p, a luciferase reporter assay was employed. As shown in Figure 7E, miR-499a-5p mimics significantly decreased the luciferase activity of the VAV3-WT-3′-UTR plasmids compared to the mimics NC group, whereas, downregulation of miR-499a-5p by ASO-miR-499a-5p notably increased the luciferase activity of the VAV3-WT-3′-UTR plasmids in 293T cells compared to the ASO-NC group. These results showed that VAV3 was a binding target of miR-499a-5p.

As shown in Figure 7F, the luciferase activity of IGF1-WT-3′-UTR significantly decreased in cells transfected with miR-576-5p mimics, whereas ASO-miR-576-5p markedly elevated the luciferase activity of IGF1-WT-3′-UTR. These results showed that IGF1 was a binding target of miR-576-5p.

## 4 Discussion

MicroRNAs (miRNAs) are potent genetic regulators, demonstrated by their ability to influence cellular pathways through interactions with multiple target genes (Diener et al., 2022). This characteristic makes the miRNAs intriguing candidates for the therapeutic applications (Diener et al., 2022). Although studies on miRNA profiles in NB are limited, some research has explored the differences in miRNA expression levels between healthy controls and brucellosis patients (Zhang et al., 2022; Budak et al., 2016). For instance, Zhang et al. observed a significant decrease in serum levels of miR-155 in brucellosis patients compared to non-brucellosis patients or healthy controls (Zhang et al., 2022). Budak et al. reported elevated levels of miR-1238-3p, and reduced levels of miR-494, miR-6069, and miR-139-3p in the blood samples from chronic brucellosis patients compared to healthy controls (Budak et al., 2016). These findings suggested that miRNAs may serve as potential biomarkers for brucellosis.

In this study, DEmiRNAs in CSF samples were screened between control and NB groups, revealing a total of 51 DEmiRNAs between two groups. These identified miRNAs may play a role in NB development. Next, to further identify NB-related miRNAs, a GSE107554 dataset comprising peripheral blood CD4 T cell samples from brucellosis patients and healthy controls was utilized as a validation cohort. Our results showed that the expression profiles of four miRNAs (hsa-miR-342-3p, hsa-miR-576-5p, hsa-miR-15b-5p and hsa-miR-499a-5p) were consistent in both the in-house and validation cohorts. Notably, NB is recognized as a complication of systemic *Brucella* infection (Mongkolrattanothai et al., 2017). Therefore, these four common DEmiRNAs identified across the in-house and validation cohorts may potentially serve as biomarkers for brucellosis, especially in NB.

The four DEmiRNAs, miR-499-5p, miR-576-5p, miR-342-3p and miR-15b-5p, have been linked to infectious diseases (Cao et al., 2023; Lu et al., 2019; Ramirez et al., 2018; Jarret et al., 2016). Chen et al. observed downregulated levels of miR-499a-5p in the peripheral blood of patients with pneumonia induced by M. pneumoniae (Chen et al., 2022). Yang et al. discovered reduced serum miR-499a-5p levels in sepsis-induced myocardial dysfunction (SIMD), and highlighted its potential as a diagnostic marker in SIMD (Yang and Wen, 2021). Conversely, Jarret et al. reported elevated levels of miR-499a-5p in Hepatitis C virus-infected liver cells (Jarret et al., 2016). These studies indicated a potential link between dysregulated miR-499a-5p and pathogen infection. Furthermore, miR-576-5p has been found to be elevated various diseases, including cancers (Chen et al., 2023; Luo et al., 2021), nonalcoholic fatty liver disease (Soronen et al., 2016)and infectious diseases (Lu et al., 2019; Ge et al., 2013). Meanwhile, evidence has shown that miR-576-5p level was notably upregulated in patients with latent tuberculosis infection (Lu et al., 2019), and in patients with pertussis (an acute respiratory infection caused by *Bacillus* pertussis) (Ge et al., 2013). These findings also indicated a potential association between miR-576-5p and pathogen infection. In this study, our results showed that miR-499a-5p and miR-576-5p levels were notably elevated in CSF samples of NB patients compared to the control group, suggesting that miR-499a-5p and miR-576-5p may serve as potential biomarkers for the diagnosis of NB.

MiR-15b-5p has also been implicated in bacterial and viral infection (Ramirez et al., 2018; Kim et al., 2020). For instance, miR-15b-5p levels were notably reduced in the lung tissue of hamsters infected with SARS-CoV-2, the virus responsible for novel coronavirus disease (COVID-19) (Kim et al., 2020). Conversely, research by Ramirez et al. revealed that *Staphylococcus aureus* could hinder wound closure and elevate miR-15b-5p levels in chronic diabetic foot ulcers (Ramirez et al., 2018). These findings suggest a dual role for miR-15b-5p, where it may either exacerbate infection or contribute to host defense and infection control. Furthermore, dysregulation of miR-342-3p has been closely linked to pathogen infections. Studies have demonstrated upregulation of miR-342-3p in various infected tissues, such as liver tissues in mice infection with P. chabaudi malaria (Al-Quraishy et al., 2012), the peripheral blood of infants infected with respiratory syncytial virus (Wang et al., 2017) and the brains from patients with sporadic Creutzfeldt-Jakob disease (Montag et al., 2009). Based on data from the in-house cohort, our results indicated a significant elevation of miR-15b-5p and miR-342-3p levels in CSF samples from patients with NB compared to the control group, suggesting these miRNAs may have potential as biomarkers for diagnosing NB.

In order to enhance the clinical utility of our findings, we assessed the diagnostic efficacy of four miRNAs and subsequently established a gene signature for the diagnosis of NB. The results of the ROC curve analysis showed that all four miRNAs exhibited promising diagnostic potential for NB in both the in-house and validation cohorts. Utilizing the LASSO regression analysis, we identified two key miRNAs (miR-576-5p and miR-499a-5p) as central to NB pathology. Subsequently, a risk score was then computed based on the levels of these miRNAs and the coefficients from LASSO regression. Notably, our results demonstrated an AUC value of 1.000 in the in-house cohort, indicating that the miRNA signature may accurately predict the diagnosis of NB patients.

MiRNAs have been demonstrated to regulate both physiological and pathological processes through enhancing mRNA degradation or suppressing target mRNA translation (Huang, 2018). Consequently, we utilized the Targetscan and miTarBase databases to predict the target genes of miR-576-5p and miR-499a-5p, respectively. Our results indicated that VAV3 was a binding target of miR-499a-5p, and IGF1 was a binding target of miR-576-5p. Previous research has revealed a link between VAV3 and inflammation and infection (Zhang et al., 2023; Roth et al., 2016), with studies showing that overexpression of VAV3 could mitigate cardiomyocyte inflammation and apoptosis in rats with myocardial infarction (Zhang et al., 2023). Additionally, Roth et al. found that deficiency of VAV genes (VAV1, VAV2 and VAV3) was associated with decreased survival rates in mice with C. albicans infection compared to wild type mice, highlighting the importance of VAV proteins in host defense against fungal infections (Roth et al., 2016). These findings suggested that VAV3 may possess anti-infective and anti-inflammatory properties. Our study revealed a significant elevation in miR-499a-5p levels and a marked reduction in VAV3 levels in CSF samples of NB patients compared to the control group. Based on these results, we hypothesize that miR-499a-5p may aggravate NB infection associated inflammatory response by targeting VAV3; however, further investigation is required to validate this assumption in the future.

IGF1 plays a crucial role in the development and cell differentiation of the nervous system (Benarroch, 2012). It is produced by various cells and can be detected in biological fluids such as CSF (Salehi et al., 2008). Evidence has shown that IGF1 deficiency was associated with the progression of nervous system diseases, such as Down’s syndrome and Parkinson (Herrera et al., 2024; Araya et al., 2022). Significantly, IGF1 has been shown to offer protection against central catecholaminergic neuronal injury induced by lipopolysaccharide (LPS) exposure through inhibition of microglia activation (Tien et al., 2017). These findings highlight the neuroprotective role of IGF1 in nervous system diseases. Our results suggested that miR-576-5p levels were notably elevated, but IGF1 levels were significantly reduced in CSF samples of NB patients compared to the control group. This observation leads us to speculate that miR-499a-5p may affect the nervous system and contribute to NB development by targeting IGF1; however, further investigation is required to confirm this hypothesis in future studies.

This article still has some limitations that need to be studied in the future. First, in this study, we found that miR-499a-5p and miR-576-5p levels were notably elevated in CSF samples of NB patients, indicating a potential association between the occurrence of NB and alterations in the expression of these miRNAs. Additionally, the ROC results showed that both miR-499a-5p and miR-576-5p both exhibit significant diagnostic value for NB. However, determining whether a miRNA serves as a specific biomarker for a particular disease, as opposed to others, is a complex task. Future validation experiments involving diverse patient cohorts and sample types are essential to confirm the disease-specific association of changes in miRNA expression. Thus, in future studies, we intend to broaden our control group to encompass a wider array of neuroinflammation-related diseases, thereby confirming the diagnostic specificity of these miRNAs in NB. Second, we found that the levels of VAV3 and IGF1 were notably decreased in CSF sample of NB patients. Tthrough literature research, we found that VAV3 may exhibit anti-infective and anti-inflammatory properties, and IGF1 may play a neuroprotective role in nervous system diseases. However, the functional roles of VAV3 and IGF1 in NB remain unexplored. Thus, further investigation is warranted to elucidate the functional roles of VAV3 and IGF1 in NB. Third, although we tried to include as many samples as possible, the current sample size still needs to be further expanded. In the near future, a larger sample size would be employed to improve the robustness of our study.

To summarize, the future direction of work should focus on exploring the deeper mechanisms underlying the onset of NB, meanwhile further optimizing the study design, such as including paired blood-CSF samples to enhance the reliability and comprehensiveness of miRNA analysis.

## 5 Conclusion

Collectively, the miRNA diagnostic signature identified in this study may show promise for diagnosing NB. Additionally, utilizing miRNAs (miR-499a-5p and miR-576-5p) as biomarkers for NB may play a significant role in personalized medicine.

## Data Availability

Data supporting the conclusions of this study are openly available at https://www.ncbi.nlm.nih.gov/search/all/?term=PRJNA1272809, reference number PRJNA1272809.
