# Supplementary figures and images for: Identification of potential miRNA biomarkers for neurobrucellosis diagnosis
Source: Front Physiol. 2025 Jun 23;16:1463597. doi: 10.3389/fphys.2025.1463597 (PMC12230071; doi:10.3389/fphys.2025.1463597)

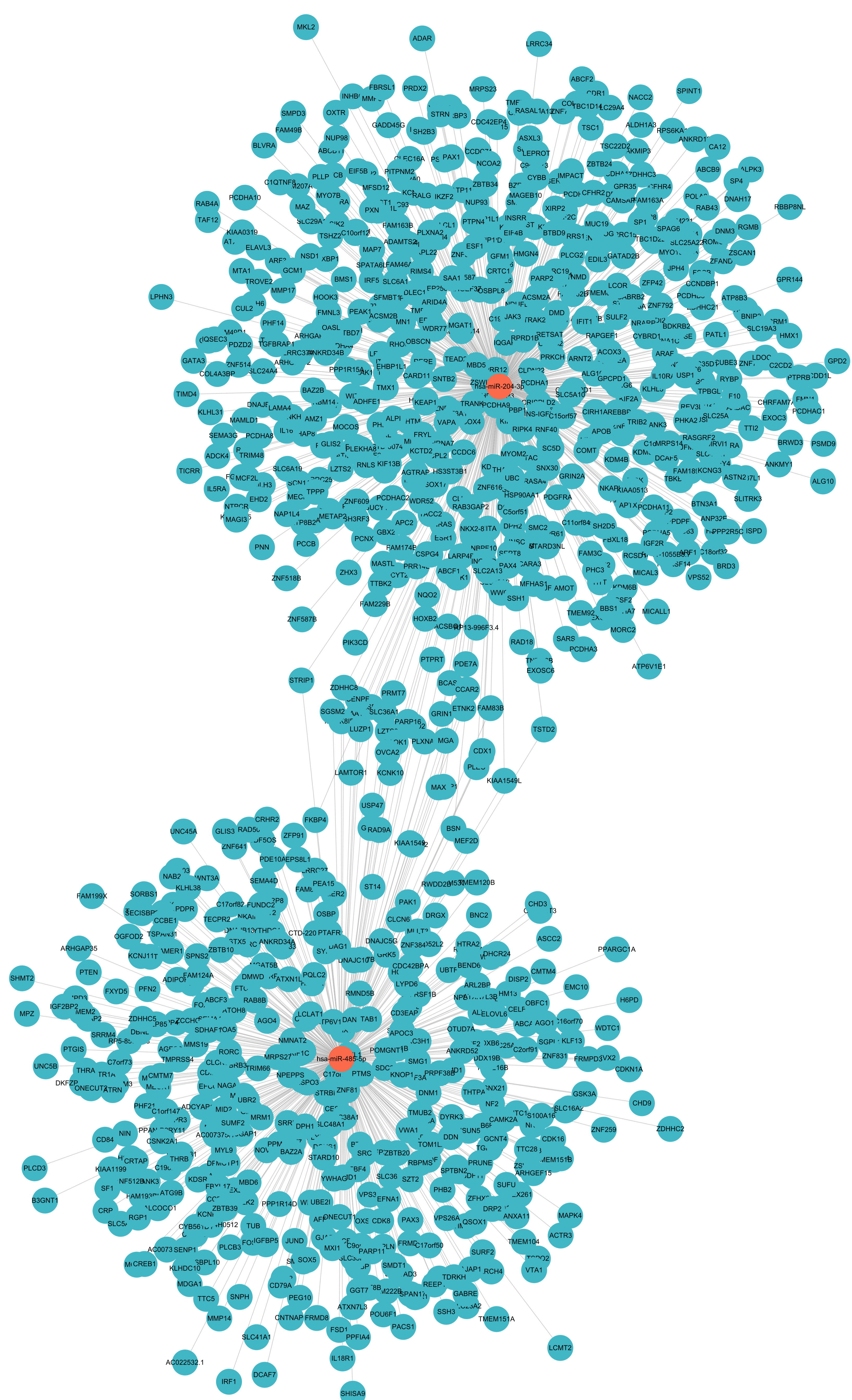

Supplement: Supplementary file 1 [file DataSheet2.PDF]

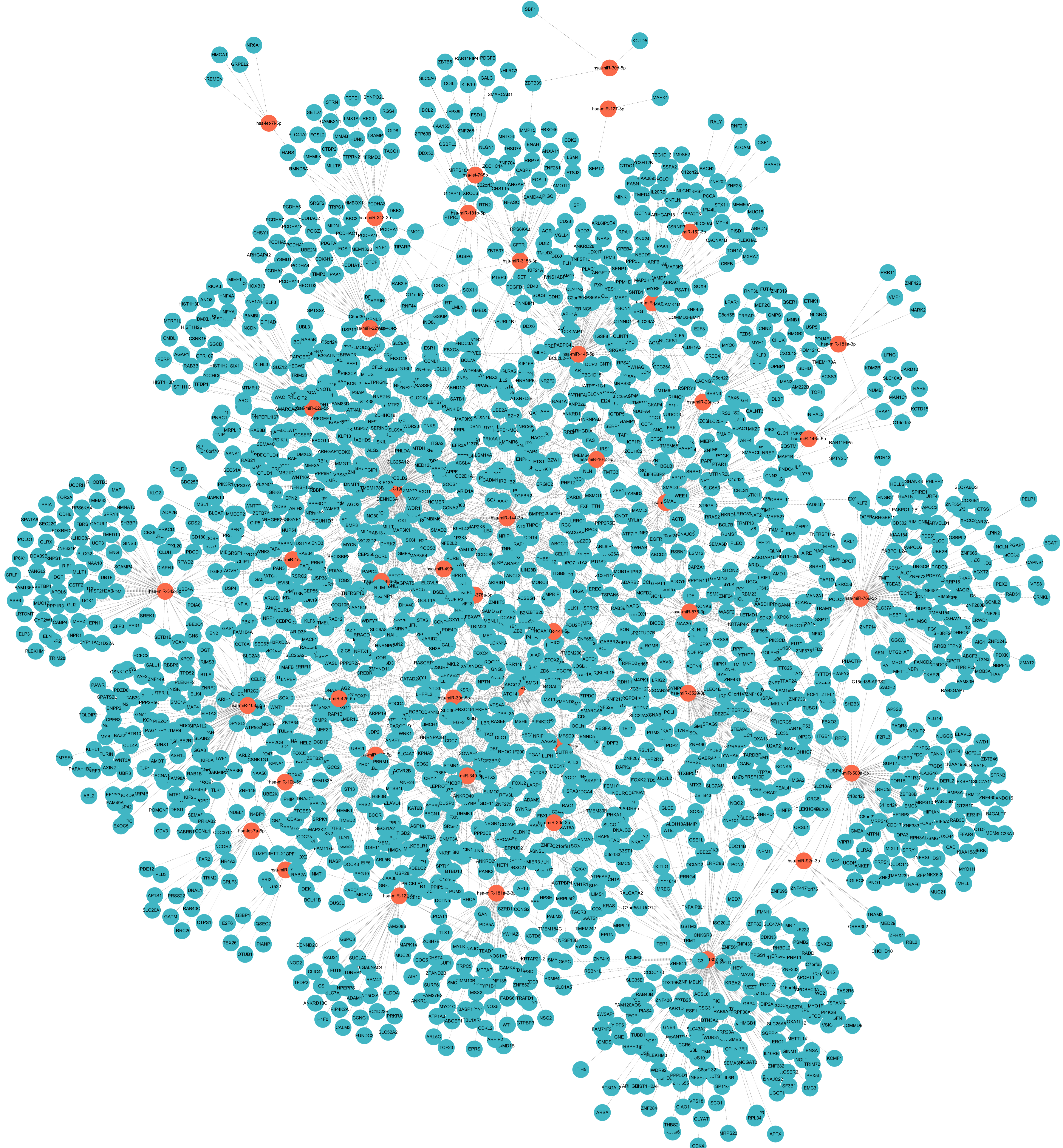

Supplement: Supplementary file 5 [file DataSheet1.PDF]
